# Damage control resuscitation in resource-diverse settings: a survey-based comparison of trauma care practices worldwide

**DOI:** 10.1590/acb412826

**Published:** 2026-06-29

**Authors:** Leticia Stefani Pacheco, Rafael Dib Possiedi, Thomas Michael Scalea, Marcelo Augusto Fontenelle Ribeiro

**Affiliations:** 1 Pontifícia Universidade Católica de São Paulo – Health Sciences and Medical School of Sorocaba – Department of Surgery – Sorocaba (SP) – Brazil.; 2 University of Maryland – School of Medicine – Department of Surgery – Baltimore (MD) – United States.; 3 Doctors Without Borders – Department of Surgery – Brussels – Belgium.; 4 UK-MED – Department of Surgery – Manchester – United Kingdom.

**Keywords:** Blood Transfusion, Trauma Centers, Multiple Trauma, Shock, Hemorrhagic, Resuscitation

## Abstract

**Purpose:**

Trauma is a major global cause of death, with uncontrolled hemorrhage as its leading preventable cause. Damage control resuscitation (DCR) is a cornerstone strategy for hemorrhagic shock, yet implementation varies by resource availability. This study compared DCR practices between high-income countries (HICs) and low- and middle-income countries (LMICs), predominantly represented by institutions from Latin America, particularly Brazil.

**Methods:**

A cross-sectional survey of trauma care providers was conducted between 2024 and 2025, assessing DCR practices, resuscitation strategies, and adjunctive therapies. Data from a Brazilian national survey were incorporated into the LMIC cohort. Countries were classified using World Bank 2024 income criteria, and comparisons were analyzed using hierarchical logistic regression with adjustment for multiple comparisons.

**Results:**

Massive transfusion protocol availability result was significantly lower in LMICs than in HICs (47.4 *vs*. 89.1%; OR = 0.11, 95%CI 0.02–0.62; *p*(BH) < 0.001). LMICs also had reduced availability of fresh frozen plasma (OR = 0.04; *p*(BH) = 0.035), balanced crystalloids (OR = 0.21; *p*(BH) < 0.001), viscoelastic coagulation monitoring (TEG/ROTEM; OR = 0.04; *p*(BH) 0.001), prothrombin complex concentrate (OR = 0.01; *p*(BH) < 0.001), fibrinogen concentrate (OR = 0.10; *p*(BH) < 0.001), rapid infusers (OR = 0.04; *p*(BH) < 0.001), invasive temperature monitoring (OR = 0.02; *p*(BH) < 0.001), calcium replacement (OR = 0.17; *p*(BH) = 0.002), and active rewarming systems (OR = 0.21; *p*(BH) = 0.035). Whole blood use was lower in LMICs (5.8 vs. 17.4%) but did not reach statistical significance in the primary model (OR = 0.55, 95%CI 0.12–2.45). Tranexamic acid availability was nearly universal and did not differ between settings (96.1 *vs*. 97.8%).

**Conclusion:**

Although DCR principles are widely recognized, substantial disparities persist in access to critical resuscitation resources across income settings.

## Introduction

Trauma accounts for approximately one in ten deaths worldwide, with uncontrolled hemorrhage representing the leading preventable cause and responsible for an estimated 1.5 million deaths annually^1^. Damage control resuscitation (DCR) is a widely adopted strategy for the management of severely injured patients with hypovolemic shock, aiming to reduce morbidity and mortality through early hemorrhage control, restriction of crystalloid administration, and balanced blood product resuscitation^2^. Central to DCR is the prioritization of bleeding control, reflected in the shift from the traditional airway–breathing–circulation sequence to a circulation-first approach, first articulated in 2007^3^.

Volume replacement remains a core component of DCR and has evolved substantially over the past two decades, initially shaped by military experience and subsequently translated into civilian trauma care. The introduction of massive transfusion protocols (MTPs) reduced excessive crystalloid and colloid use while promoting early, balanced administration of blood products. More recently, whole blood, particularly low-titer group O whole blood, has been increasingly incorporated into both prehospital and in-hospital resuscitation strategies to optimize early physiologic stabilization^4^. Compared with component therapy, whole blood provides more efficient oxygen delivery and hemostatic support, and its early use has been associated with reductions in 24-hour and 30-day mortality, as well as decreased overall transfusion requirements^5,6^. These benefits appear consistent across both prehospital and in-hospital settings, underscoring the importance of timely administration^4,7^.

In parallel with the adoption of whole blood, other components of DCR have become increasingly standardized, including early hemorrhage control, calcium, and tranexamic acid administration, fibrinogen replacement, and coagulation-guided resuscitation using viscoelastic testing such as thromboelastography or rotational thromboelastometry (TEG/ROTEM)^2,8^. Contemporary DCR recommendations from the American Association for the Surgery of Trauma and the American College of Surgeons^9^, as well as prior national data from Brazil^10^, highlight both the growing global acceptance of DCR principles and the potential for variability in their implementation.

In this context, the present study compared DCR practices across diverse trauma systems to identify structural disparities between high-income and low- and middle-income settings, addressing the limited availability of comparative global data on DCR implementation.

## Methods

This study was conducted between 2024 and 2025 using a global, anonymous survey administered to general and trauma surgeons, intensivists, anesthesiologists, and other physicians directly involved in DCR. The questionnaire comprised two domains: demographic and training characteristics, and institutional practices related to DCR. The second domain assessed the availability and activation of MTP, prioritization of a circulation-first approach, fluid strategies, permissive hypotension, adjunct therapies, hemorrhage control resources, intra-abdominal pressure monitoring, and markers used to evaluate physiological response to resuscitation. The survey was developed by a multidisciplinary trauma team, and the model was validated by the senior author of this study in a previous publication^10^. The full survey instrument, including English and Portuguese versions, is provided in the Suppl. Mat. 1 and 2. The survey was distributed electronically through professional trauma and surgical networks (Worldwide trauma surgeons WhatsApp group), institutional mailing lists, and direct invitations to eligible physicians. Participation was voluntary, and informed consent was obtained electronically prior to survey completion. As this study involved an anonymous survey of healthcare professionals without collection of patient-level or identifiable data, formal institutional review board approval was not required.

Responses were classified as originating from high-income countries (HICs) or low- and middle-income countries (LMICs) according to the 2024 World Bank Gross National Income criteria. To enhance LMIC representation, data collected in 2024 from a previously published Brazilian national survey^10^ were incorporated into the LMIC group, as the same survey instrument and methodology were used without modification. Responses lacking country of practice or key institutional information were excluded. Statistical analyses compared DCR practices between income groups.

### Statistical analysis

Data analysis occurred in two stages. First, exploratory descriptive statistics were applied to all responses. Second, to compare trauma practices between income groups, we used hierarchical logistic regression with a random intercept by country and a fixed HIC *versus* LMIC contrast, accounting for clustering due to the large Brazilian sample, in accordance with established multilevel modeling approaches^11,12^.

For each dichotomous outcome, a logit model with a random country intercept and income group as the fixed predictor was applied, yielding cluster-adjusted *odds ratio* (OR) and 95% confidence interval (95%CI). This approach accounts for within-country correlation without individual-level covariate adjustment. In instances of separation, very large OR and non-finite upper 95%CI were interpreted as systematic presence or absence. Numerical instability was addressed using a generalized linear model with country-clustered robust standard errors.

To assess robustness, we performed two sensitivity analyses (excluding Brazil and LOCO) and adjusted *p*-values for multiple outcomes using the Benjamini–Hochberg method, reported as *p*(BH).

All analyses were performed in R (version 4.5.1) using ggplot2, ggpubr, and ggstatsplot for visualization^13-16^. Differences between HIC and LMIC were tested with hierarchical logistic regression (glmer) in lme4, reporting OR and 95%CI^17^. When separation occurred, a logistic GLM with clustered robust errors (vcovCL) via lmtest and sandwich was applied^18-20^. Multiple comparisons were corrected using the Benjamini–Hochberg method, reported as *p*(BH)^21^.

## Results

### Respondents and institutional characteristics

A total of 200 respondents were included in the analysis, after exclusion of three participants due to insufficient information on country of practice. According to the World Bank income classification, most respondents were from LMICs (154/200, 77%), while 46/200 (23%) practiced in HICs (Suppl. Mat. 1). Participants represented a wide range of countries, with LMIC responses predominantly from Latin America, particularly Brazil, whereas HIC respondents were mainly from North America, Europe, and the Middle East (Suppl. Mat. 1). Most participants were trained in general surgery, with a high proportion reporting additional training in trauma, critical care, or both, and over half of respondents in both groups had more than 10 years of professional experience (Suppl. Mat. 1). Regarding institutional setting, public hospitals predominated overall (140/200, 70%), particularly in LMICs (118/154, 76.62%) compared with HICs (22/46, 47.83%), while academic hospitals were proportionally more frequent among HIC respondents (26/46, 56.52%) than in LMICs (62/154, 40.26%). Detailed country distribution, training background, years of practice, and hospital characteristics are provided in Suppl. Mat. 1 and 2.

### Availability and activation criteria of massive transfusion protocols

The availability of a MTP differed significantly between income settings. Overall, MTP was reported as available by 57% of respondents (114/200), with substantially higher availability in HICs compared with LMICs (41/46, 89.13% *versus* 73/154, 47.4%) (Suppl. Mat. 3). In hierarchical logistic regression, respondents from LMICs had significantly lower odds of having an MTP available at their institution (OR = 0.11, 95% CI 0.02–0.62; *p*BH < 0.001), a finding that remained consistent in sensitivity analyses excluding Brazil (OR = 0.10, 95% CI 0.01–0.84) and across leave-one-country-out analyses, which consistently yielded OR below unity (Suppl. Mat. 4).

The clinical criteria used to activate the MTP were broadly similar across income groups (Suppl. Mat. 3 and 5). Common triggers included shock index > 1 (HIC 25/46, 54.3% *versus* LMIC 64/154, 41.6%), hypotension (22/46, 47.8% *versus* 43/154, 27.9%), FAST positivity (30.4 *versus* 19.5%), pelvic fracture (17.4 *versus* 15.6%), and surgeon judgment (“gut feeling”) (45.7 *versus* 34.4%). After adjustment for multiple comparisons, no individual activation criterion differed significantly between HICs and LMICs. Advanced viscoelastic testing (TEG/ROTEM) was reported more frequently as an activation trigger in HICs (5/46, 10.87%) compared with LMICs (3/154, 1.95%), whereas the response “none of the above” was more common in LMICs (24/154, 15.58%) than in HICs (1/46, 2.17%), although both findings did not reach statistical significance after correction and were characterized by wide confidence intervals (Suppl. Mat. 5). Detailed numerical results and additional analyses are presented in Suppl. Mat. 3–7.

### Transfusion strategies and volume replacement practices

The use of blood components and crystalloids for volume replacement differed significantly between income settings (Suppl. Mat. 8 and 9). Packed red blood cells were widely used in both groups (HIC 45/46, 97.83% *versus* LMIC 143/154, 92.86%), with no statistically significant difference after adjustment. In contrast, clinicians in LMICs had significantly lower odds of administering fresh frozen plasma (HIC 44/46, 95.7% *versus* LMIC 124/154, 80.5%; OR = 0.04, 95% CI 0.00–0.39; pBH = 0.035) and platelets (HIC 39/46, 84.8% *versus* LMIC 102/154, 66.2%; OR = 0.35, 95%CI 0.15–0.84; *p*BH = 0.037). The most pronounced disparity was observed in the use of balanced crystalloids, which were reported by 29/154 LMIC respondents (18.8%) compared with 24/46 in HICs (52.2%) (OR = 0.21, 95%CI 0.11–0.43; *p*BH < 0.001). No statistically significant differences were observed in the use of lactated Ringer’s solution or normal saline. The use of liquid plasma and cryoprecipitate showed a trend toward lower utilization in LMICs, although these differences did not reach statistical significance after correction.

Marked differences were also observed in transfusion strategies adopted during massive transfusion (Suppl. Mat. 10 and 11). Overall, the 1:1 red blood cell to plasma ratio was the most frequently reported strategy (164/200, 82%), followed by the 1:1:1 ratio including platelets (122/200, 61%). After adjustment for multiple comparisons, no statistically significant differences were identified between HICs and LMICs for any transfusion ratio. Whole blood transfusion was reported more frequently in HICs than in LMICs (8/46, 17.4% versus 9/154, 5.8%). Although this difference did not reach statistical significance in the primary model (OR = 0.55, 95% CI 0.12–2.45), sensitivity analyses demonstrated consistently lower odds of whole blood use in LMICs across leave-one-country-out analyses (Suppl. Mat. 11). The use of crystalloids as the primary transfusion strategy was reported exclusively in LMICs (15/154, 9.7% *versus* 0/46, 0% in HICs), though this finding was characterized by sparse data and did not remain statistically significant after correction. Expanded results and supporting analyses can be found in Suppl. Mat. 8–15.

### Adjuncts and infrastructure supporting damage control resuscitation

Marked disparities were observed in the availability of adjuncts and infrastructure supporting damage control resuscitation between income settings (Suppl. Mat. 16 and 17). Access to advanced coagulation products and monitoring tools was substantially lower in LMICs. Compared with HICs, respondents from LMICs had significantly lower odds of reporting availability of prothrombin complex concentrate (5/154, 3.25% *versus* 35/46, 76.09%; OR = 0.01, 95%CI 0.00–0.11; *p*BH < 0.001), fibrinogen concentrate (37/154, 24.03% *versus* 35/46, 76.09%; OR = 0.10, 95%CI 0.05–0.22; *p*BH < 0.001), and viscoelastic testing (TEG/ROTEM) (11/154, 7.14% *versus* 34/46, 73.91%; OR = 0.04, 95%CI 0.01–0.17; *p*BH < 0.001). Similarly, the availability of rapid infusers (17/154, 11.04% *versus* 36/46, 78.26%; OR = 0.04, 95%CI 0.01–0.18; *p*(BH) < 0.001), invasive temperature monitoring (4/154, 2.6% *versus* 32/46, 69.57%; OR = 0.02, 95%CI 0.00–0.12; *p*(BH) < 0.001), arterial blood gas analysis in the trauma bay (69/154, 44.81% *versus* 37/46, 80.43%; OR = 0.10, 95%CI 0.03–0.33; *p*(BH) < 0.001), calcium replacement (109/154, 70.78% *versus* 43/46, 93.48%; OR = 0.17, 95%CI 0.05–0.57; *p*(BH) = 0.002), and active rewarming systems (85/154, 55.19% *versus* 34/46, 73.91%; OR = 0.21, 95%CI 0.06–0.71; *p*(BH) = 0.035) was also significantly reduced in LMICs.

In contrast, the use of tranexamic acid was nearly universal and did not differ between income groups (148/154, 96.1% in LMICs *versus* 45/46, 97.83% in HICs; OR = 0.55, 95% CI 0.06–4.67; *p*BH = 1.000). Fluid warmers were reported more frequently in HICs (50 versus 0% in LMICs; 23/46 *versus* 0/154; *p*BH < 0.001). Additional descriptive and comparative analyses are provided in Suppl. Mat. 16–25.

## Discussion

Damage control resuscitation principles are widely recognized and endorsed across trauma systems worldwide. However, our findings demonstrate substantial structural disparities between HICs and LMICs in the availability of key resources required to implement evidence-based DCR strategies. These differences were most pronounced in access to MTP, blood products, coagulation monitoring, and adjunctive hemorrhage control technologies.

Following American Association for the Surgery of Trauma and American College of Surgeons’ recommendations, DCR emphasizes rapid hemorrhage control, antifibrinolytics, restricted crystalloid use, “blood for blood” resuscitation, and permissive hypotension^2,9,10,22,23^. Our findings revealed that although these principles are widely recognized, their implementation differs substantially across income settings, reflecting uneven availability of blood products, monitoring tools, and hemorrhage control resources. These discrepancies highlight persistent structural challenges that may influence the delivery of optimal trauma care worldwide.

Crystalloids were historically favored due to low cost and logistical accessibility, but excessive administration is associated with visceral edema, abdominal compartment syndrome, acute respiratory distress syndrome, dilutional coagulopathy, and worsened bleeding^22,24,25^. In prehospital settings, crystalloids remain an option when blood products are unavailable, but should be delivered cautiously in small, controlled volumes^26^. In our study, although basic fluids were used similarly across groups, LMIC respondents reported significantly lower access to balanced crystalloids. It is important to note that our study captures reported availability and choice of resuscitation fluids rather than administered volumes or fluid-related adverse outcomes.

Our results also revealed marked disparities in the availability of MTP. Although activation criteria did not differ significantly, institutions in LMICs reported much lower availability. MTP activation is a cornerstone of contemporary hemorrhage management and has been associated with improved outcomes in prior clinical studies. The survival benefit has been attributed to multiple factors, including the use of appropriate blood product ratios, earlier initiation of transfusion, and broader system improvements that facilitate coordinated hemorrhage control. Evidence from a planned sub analysis of the PROPPR trial demonstrated that each 1 minute delay in MTP activation was associated with a 5% increase in mortality, and a recent meta-analysis of more than 3,000 patients confirmed a significant reduction in overall mortality with MTP implementation^9^. Most participants supported the ‘Circulation first’ principle, aligning with evidence that emphasizes early bleeding control and hemodynamic stabilization in hypovolemic trauma patients^10,27^.

Brill et al.^6^ report that, among a broad cohort of trauma patients presenting with hemorrhagic shock, whole blood transfusion was associated with a 60% improvement in survival at 30 days. The survival effect extended across multiple injury severity strata, though it was most pronounced in individuals with ISS values between 5 and 40. In addition, patients treated with whole blood required 7% fewer blood products during the first 24 hours. These observations aligned with the authors’ original hypothesis that whole blood would yield higher survival while reducing overall transfusion volume when compared with the use of component therapy alone^6^.

Shackelford et al.^28^ identify whole blood as the preferred resuscitation product for hemorrhagic shock at all roles of care, recommending low titer O whole blood whenever available and within 30 minutes of wounding. When whole blood cannot be provided, component therapy should follow the order of preference outlined in the Tactical Combat Casualty Care Guidelines. Their recommendations also underscore the logistical requirements for whole blood programs, including donor screening, unit prescreening, tracking systems, and personnel training^28^.

Our findings demonstrated significantly lower use of whole blood and balanced component therapy in LMICs. This disparity likely reflects structural barriers to implementing whole blood programs, including the need for coordinated blood banking systems, reliable donor screening, cold chain logistics, regulatory oversight, and trained transfusion personnel. These differences persisted across robustness analyses. Although our study was not designed to assess patient-level clinical outcomes, these findings highlight important structural and logistical barriers to the implementation of whole blood–based resuscitation strategies. It is also important to note that most LMIC respondents in our sample were from middle-income countries in Latin America, particularly Brazil. Therefore, the disparities observed here may underestimate the true global gap in access to DCR resources, which may be even greater in lower-income regions such as parts of Sub-Saharan Africa.

Calcium supplementation is another essential component of DCR, recommended early and repeated every four units of blood products^9^. Our study found significantly reduced calcium use in LMICs, along with limited access to thromboelastometry, rapid infusers, and invasive temperature monitoring. These deficits may limit the ability of some institutions to rapidly detect and correct trauma-induced coagulopathy. In contrast, tranexamic acid use was similar across groups, reflecting its low cost, high availability, and strong evidence of mortality benefit when administered early in a standard dosing regimen.

Thromboelastography and rotational thromboelastometry enable real-time assessment of trauma-induced coagulopathy and support goal-directed resuscitation, particularly through early identification of fibrinogen depletion and impaired clot formation^8^. Consistent with this, our study demonstrated marked disparities in access to TEG/ROTEM in LMICs, which may limit the ability to implement coagulation-guided resuscitation strategies and the targeted use of adjuncts such as fibrinogen and prothrombin complex concentrate.

In the present study, vasopressin availability was considerably lower in LMIC settings. Although vasopressin has been investigated as an adjunct in hemorrhagic shock resuscitation, limited access to this agent may restrict the range of pharmacologic options available for managing refractory shock in some institutions. This disparity further illustrates how differences in resource availability may influence the implementation of adjunctive resuscitation strategies across trauma systems.

Hemorrhage control methods also differed substantially between the groups. Hemostatic dressings outperform standard gauze, particularly in preperitoneal pelvic packing, and may reduce transfusion needs. Tourniquets improve survival in severe extremity injury with low complication rates, while junctional tourniquets show promise for junctional bleeding, though long-term outcomes remain understudied^29^,^30^. Our findings showed significant disparities in access to hemostatic dressings, pelvic binders, thoracotomy kits, dedicated trauma ultrasound, and REBOA. Multiple organizations caution that REBOA should only be used by trained providers within advanced systems and with strict time limits, yet the AAST REBOA study group reported improved survival in pelvic hemorrhage compared with thoracotomy, although confounding limits definitive conclusions^31-33^. LMICs in our sample demonstrated markedly reduced access to REBOA and pelvic binders, highlighting structural gaps in the availability of technologies used to manage non-compressible hemorrhage.

Overall, our results demonstrated consistent and meaningful disparities in trauma resuscitation resources between HICs and LMICs. These gaps may limit the ability of some institutions to fully implement evidence-based DCR strategies and are consistent with broader global disparities in trauma system resources.

## Conclusion

Hospitals adapt DCR protocols to their available resources, resulting in substantial heterogeneity in practice patterns across income settings. Our findings demonstrated that disparities are most pronounced in access to MTP, blood products, coagulation monitoring, and key adjunctive technologies. These differences reflect persistent global variability in trauma care infrastructure and resource availability. Addressing these gaps through targeted training, system strengthening, and context-appropriate investment is essential to support the implementation of evidence-based resuscitation strategies, particularly in lower-resource settings.

### Study limitations

As a voluntary online survey distributed through professional networks, this study is subject to potential selection and response bias. Additionally, the results relied on self-reported practices, which may differ from actual bedside implementation in some institutions. The convenience sample, predominantly composed of respondents from Brazil, means that LMIC findings largely reflect an upper–middle-income setting. Additionally, the number of respondents from HIC was relatively small (n = 46), which may not fully capture the heterogeneity of trauma systems across HIC settings, such as differences between North America, Western Europe, and other high-income regions. Sensitivity analyses (LOCO and OR_noBR) helped quantify this influence, showing that while the magnitude of OR varied, the direction and significance of key disparities were mostly unchanged. As such, the results may underestimate challenges faced by LMICs. The study design identified strong associations between national income level and clinical practices but cannot establish causality, as income serves as a proxy for multiple intertwined factors, including infrastructure, training, financing, and supply chains. Finally, findings related to very rare items should be interpreted cautiously, as extreme OR may reflect the presence or absence of practice rather than a precise estimate of effect size.

## Data Availability

The supplementary materials (including tables) supporting the findings of this study are deposited in the Zenodo repository. These resources are available through the following DOIs: 10.5281/zenodo.20057895 (Supplementary Tables 1-2), 10.5281/zenodo.20058019 (Supplementary Tables 3-7), 10.5281/zenodo.20058062 (Supplementary Tables 8-15), and 10.5281/zenodo.20058116 (Supplementary Tables 16-25).

## References

[1] Richards JE, Stein DM, Scalea TM (2024). Damage control resuscitation in traumatic hemorrhage: it is more than fixing the holes and filling the tank. Anesthesiology.

[2] Lammers DT, Holcomb JB (2023). Damage control resuscitation in adult trauma patients: What you need to know. J Trauma Acute Care Surg.

[3] Ferrada P, Ferrada R, Jacobs L, Duchesne J, Ghio M, Joseph B, Taghavi S, Qasim ZA, Zakrison T, Brenner M, Dissanaike S, Feliciano D (2024). Prioritizing circulation to improve outcomes for patients with exsanguinating injury: a literature review and techniques to help clinicians achieve bleeding control. J Am Coll Surg.

[4] Holcomb JB, Butler FK, Schreiber MA, Taylor AL, Riggs LE, Krohmer JR, Dorlac WC, Jenkins DH, Cox DB, Beckett AN, O’Connor KC, Gurney JM (2024). Making blood immediately available in emergencies. Transfusion.

[5] Gurney JM, Staudt AM, Del Junco DJ, Shackelford SA, Mann-Salinas EA, Cap AP, Spinella PC, Martin MJ (2022). Whole blood at the tip of the spear: A retrospective cohort analysis of warm fresh whole blood resuscitation versus component therapy in severely injured combat casualties. Surgery.

[6] Brill JB, Tang B, Hatton G, Mueck KM, McCoy CC, Kao LS, Cotton BA (2022). Impact of incorporating whole blood into hemorrhagic shock resuscitation: analysis of 1,377 consecutive trauma patients receiving emergency-release uncrossmatched blood products. J Am Coll Surg.

[7] Levy MJ, Garfinkel EM, May R, Cohn E, Tillett Z, Wend C, Sikorksi RA, Troncoso R, Jenkins JL, Chizmar TP, Margolis AM (2024). Implementation of a prehospital whole blood program: Lessons learned. J Am Coll Emerg Physicians Open.

[8] Brill JB, Brenner M, Duchesne J, Roberts D, Ferrada P, Horer T, Kauvar D, Khan M, Kirkpatrick A, Ordonez C, Perreira B, Priouzram A, Cotton BA (2021). The role of TEG and ROTEM in damage control resuscitation. Shock.

[9] LaGrone LN, Stein D, Cribari C, Kaups K, Harris C, Miller AN, Smith B, Dutton R, Bulger E, Napolitano LM (2024). American Association for the Surgery of Trauma/American College of Surgeons Committee on Trauma: Clinical protocol for damage-control resuscitation for the adult trauma patient. J Trauma Acute Care Surg.

[10] Ribeiro MAF, Pacheco LS, Duchesne JC, Parreira JG, Mohseni S (2025). Damage control resuscitation: how it’s done and where we can improve. A view of the Brazilian reality according to trauma professionals. Rev Col Bras Cir.

[11] Puente-Palacios KE, Laros JA (2009). Análise multinível: contribuições para estudos sobre efeito do contexto social no comportamento individual. Estud Psicol.

[12] Wong GY, Mason WM (1985). The hierarchical logistic regression model for multilevel analysis. J Am Stat Assoc.

[13] Kassambara A ggpubr: ‘ggplot2’ Based Publication Ready Plots.

[14] Patil I (2021). Visualizations with statistical details: The “ggstatsplot” approach. JOSS.

[15] R Core Team (2024). R: The R Project for Statistical Computing.

[16] Wickham H. (2016). ggplot2: elegant graphics for data analysis.

[17] Bates D, Mächler M, Bolker B, Walker S (2015). Fitting linear mixed-effects models using lme4. J Stat Soft.

[18] Zeileis A, Hothorn T (2002). Diagnostic checking in regression relationships. R News.

[19] Zeileis A (2006). Object-oriented computation of sandwich estimators. J Stat Softw.

[20] Zeileis A, Köll S, Graham N (2020). Various versatile variances: An object-oriented implementation of clustered covariances in R. J Stat Softw.

[21] Chen SY, Feng Z, Yi X (2017). A general introduction to adjustment for multiple comparisons. J Thorac Dis.

[22] Leibner E, Andreae M, Galvagno SM, Scalea T (2020). Damage control resuscitation. Clin Exp Emerg Med.

[23] Salamea-Molina JC, Himmler AN, Valencia-Angel LI, Ordoñez CA, Parra MW, Caicedo Y, Guzmán-Rodríguez M, Orlas C, Granados M, Macia C, García A, Serna JJ, Badiel M, Puyana JC (2020). Whole blood for blood loss: hemostatic resuscitation in damage control. Colomb Med (Cali).

[24] Russell RT, Leeper CM, Spinella PC (2023). Damage-control resuscitation in pediatric trauma: What you need to know. J Trauma Acute Care Surg.

[25] Ordoñez CA, Parra M, Serna JJ, Rodriguez F, Garcia A, Salcedo A, Caicedo Y, Padilla N, Pino LF, González Hadad A, Herrera MA, Millán Lozano M, Quintero L, Hernandez F, Ferrada R, Brenner M, Rasmussen T, Scalea T, Ivatury R, Holcomb J (2020). Damage control resuscitation: REBOA as the new fourth pillar. Colomb Med.

[26] Melendez JJ, Caicedo Y, Guzman M, Serna JJ, Ordoñez J, Angamarca E, Garcia A, Pino LF, Quintero L, Parra M, Ordoñez CA (2020). Prehospital damage control: the management of volume, temperature… and bleeding!. Colomb Med.

[27] Ferrada P, Manzano-Nunez R, Lopez-Castilla V, Orlas C, García AF, Ordonez CA, Dubose JJ (2019). Meta-analysis of post-intubation hypotension: a plea to consider circulation first in hypovolemic patients. Am Surg.

[28] Shackelford SA, Gurney JM, Taylor AL, Keenan S, Corley JB, Cunningham CW, Drew BG, Jensen SD, Kotwal RS, Montgomery HR, Nance ET, Remley MA, Cap AP, Joint Trauma System Defense Committee on Trauma, Armed Services Blood Program (2021). Joint Trauma System, Defense Committee on Trauma, and Armed Services Blood Program consensus statement on whole blood. Transfusion.

[29] Granville-Chapman J, Jacobs N, Midwinter MJ (2011). Pre-hospital haemostatic dressings: a systematic review. Injury.

[30] Inaba K, Siboni S, Resnick S, Zhu J, Wong MD, Haltmeier T, Benjamin E, Demetriades D (2015). Tourniquet use for civilian extremity trauma. J Trauma Acute Care Surg.

[31] Bini JK, Hardman C, Morrison J, Scalea TM, Moore LJ, Podbielski JM, Inaba K, Piccinini A, Kauvar DS, Cannon J, Spalding C, Fox C, Moore E, DuBose JJ, AAST AORTA Study Group (2022). Survival benefit for pelvic trauma patients undergoing Resuscitative Endovascular Balloon Occlusion of the Aorta: Results of the AAST Aortic Occlusion for Resuscitation in Trauma Acute Care Surgery (AORTA) Registry. Injury.

[32] Nunez RM, Naranjo MP, Foianini E, Ferrada P, Rincon E, García-Perdomo HA, Burbano P, Herrera JP, García AF, Ordoñez CA (2017). A meta-analysis of resuscitative endovascular balloon occlusion of the aorta (REBOA) or open aortic cross-clamping by resuscitative thoracotomy in non-compressible torso hemorrhage patients. World J Emerg Surg.

[33] Brenner M, Bulger EM, Perina DG, Henry S, Kang CS, Rotondo MF, Chang MC, Weireter LJ, Coburn M, Winchell RJ, Stewart RM (2018). Joint statement from the American College of Surgeons Committee on Trauma (ACS COT) and the American College of Emergency Physicians (ACEP) regarding the clinical use of Resuscitative Endovascular Balloon Occlusion of the Aorta (REBOA). Trauma Surg Acute Care Open.

